# A bibliometric and visualization analysis on the association between chronic exposure to fine particulate matter and cancer risk

**DOI:** 10.3389/fpubh.2022.1039078

**Published:** 2022-12-05

**Authors:** Xuman Luo, Qiuping Yang, Daitian Zheng, Huiting Tian, Lingzhi Chen, Jinyao Wu, Zeqi Ji, Yexi Chen, Zhiyang Li

**Affiliations:** Department of Thyroid, Breast, and Hernia Surgery, General Surgery, The Second Affiliated Hospital of Shantou University Medical College, Shantou, Guangdong, China

**Keywords:** bibliometric analysis, association, cancer, fine particulate matter, exposure

## Abstract

**Introduction:**

As one of the major pollutants in ambient air pollution, fine particulate matter (PM_2.5_) has attracted public attention. A large body of laboratory and epidemiological research has shown that PM_2.5_ exposure is harmful to human health.

**Methods:**

To investigate its association with the commonly observed PM-related cancer, a bibliometric study was performed on related publications from 2012 to 2021 from a macroscopic perspective with the help of the Web of Science database and scientometric software VOSviewer, CiteSpace V, HistCite, and Biblioshiny.

**Results:**

The results indicated that of the 1,948 enrolled documents, scientific productions increased steadily and peaked in 2020 with 348 publications. The most prolific authors, journals, organizations, and countries were Raaschou-Nielsen O, *Science of the Total Environment*, the Chinese Academy of Sciences, and China, respectively. The top five keywords in frequency order were “air pollution,” “particulate matter,” “lung cancer,” “exposure,” and “mortality.”

**Discussion:**

The toxic mechanism of carcinogenicity was explained and is worthy of further investigation. China and the US collaborated most closely, and it is hoped the two countries can strengthen their collaboration to combat air pollution. There is also a need to identify the components of PM_2.5_ and refine the models to assess the global burden of disease attributed to PM_2.5_ exposure.

## Introduction

With the acceleration of industrialization and urbanization, air pollution has become a pressing problem. Notwithstanding action taken to improve air quality, it is still recognized as the largest global environmental cause of diseases and premature mortality, as reported by Copat et al. (1). In the context of the COVID-19 pandemic, fine particulate matter, namely PM_2.5_ (aerodynamic diameter ≤2.5 μm), has been attracting more and more attention, as the latest evidence suggests it may not only serve as a carrier for virus infection but also exacerbate the impact on humans (1). Based on the Global Burden of Diseases, Injuries, and Risk Factors Study 2019, the particulate pollution burden was 44.6% higher compared to what it was in 2017 (2). As the main air pollutant, many research studies have confirmed that chronic exposure to ambient PM_2.5_ was a factor that severely affected human health, especially, but not limited to, cardiovascular and respiratory diseases such as cardiac arrhythmias and COPD (3, 4). Among various detrimental effects, PM-related cancer was commonly observed and required further research due to its high mortality and growing burden. For example, lung cancer, which accounted for 18% of global cancer deaths in 2020 (5), had relative risks of incidence and mortality, respectively, of 1.08 and 1.11 under long-term exposure to PM_2.5_ (6). It was estimated that among men in East Asia, PM_2.5_ was responsible for 42.2% of lung cancer deaths (7). Declared by the IARC as a Class I carcinogen (8), two mainstream biological mechanisms exist.

For one thing, PM-induced oxidative stress can act on epithelial cells, producing reactive oxygen species (ROS) that can further damage DNA, proteins, and lipids. For another, inflammation, directly or indirectly evoked by PM_2.5_, can cause the secondary emergence of chemokines and cytokines, which can trigger tumor growth and metastasis (angiogenesis), allowing malignant cells to invade epithelial cells and thus guaranteeing the survival of these malignant cells when they metastasize to distant organs (9). An increasing number of publications are emerging on PM_2.5_ exposure and cancer, including the carcinogenicity and mechanism; however, the correlation remains poorly understood.

Bibliometrics, developed by Alan Pritchard in England in 1969 (10), is an emerging and popular discipline integrating mathematics, statistics, and other measurement methods to calculate and analyze distribution structure, quantitative relationships, change patterns, and quantitative management of literature information based on huge scholarly publications, which have been applied to many fields, including medicine (11). With sparse studies using bibliometrics to probe the relationship between PM_2.5_ and cancer, this review aims to explore the research progress and future directions on this topic from a macroscopic perspective by searching papers in the Web of Science database and analyzing them using bibliometric methods. Hopefully, it can accurately assess the current status by organizing large volumes of information and providing useful ideas for future researchers.

## Materials and methods

### Data source

Data collection was performed on October 12, 2022, in the Web of Science (WoS) core collection database, which has a reputation as an original citation index and contains substantial peer-reviewed scientific productions, including SCI-EXPANDED (2003–present), SSCI (2003–present), A&HCI (2003–present), ESCI (2017–present), CCR-EXPANDED (1985–present), and IC (1993–present).

PubMed and its MeSH database were utilized to screen the subject terms and entry terms related to PM_2.5_ and cancer. To ensure the recall ratio, a rough search of related articles was also conducted. Their titles and abstracts were read to extract keywords that met our requirements as much as possible. Therefore, we devised a search strategy: #1, TS = (“PM_2.5_”) OR TS = (“fine particulate matter”) OR TS = (“particulate matter 2.5”); #2, TS = (cancer) OR TS = (tumor) OR TS = (neoplasm) OR TS = (neoplasia) OR TS = (malignancy); #3, and #1 AND #2.

The above queries yielded a total of 2,562 outputs. After setting a limit on the publication year from January 2012 to December 2021, only reviews and articles were extracted to offer more credible results. Filtered to be in the English language, we excluded one retracted article and 60 documents that were published in 2022. A total of 1,948 documents were derived in the format of “plain text file” with the record content of “full record and cited references” and then renamed “download ^*^.txt” so that CiteSpace could recognize them (Supplementary Figure S1).

### Data analysis

The bibliometric analysis combined objective procedures like performance analysis with subjective ones such as thematic analysis to assess article performance, collaboration among authors, institutions, and countries, and recent research hotspots with future directions (12). Bibliometric software like VOSviewer, HistCite, CiteSpace, and Gephi makes the aforementioned analysis possible and effective; it is widely used in this field. However, there was no census on which software was the best. Each piece of software was chosen according to its respective advantages and features. VOSviewer 1.6.18 and Biblioshiny were mainly applied in this research, with HistCite Pro 1.2.1 and CiteSpace V 5.8 R3 selected as supplements.

Biblioshiny, an online analytical platform using R Studio, offered information on annual scientific productions and average citations per year (13). The H index and M index of the 10 most productive authors were provided as measurements of the impact of academic researchers (14). The above-referenced data were shown in three-line tables or figures in Microsoft Word 2019 and Microsoft PowerPoint 2019.

The impact factor (IF) in 2022 and the JCR partition of 10 top journals were accessed in the Web of Science's Incites Journal Citation Reports.

VOSviewer, developed by Leiden University, adept at creating, visualizing, and clustering networks contributed to performance analysis including the top 10 prolific authors, institutions, journals, and countries and according to citations of each item and was used to conduct (1) co-citation analysis: building the top 18 most co-cited references network based on co-occurrence relationship of citation. Namely, if A cited B and C at the same time, B and C share a relationship of co-citation, which varies as time goes by. Therefore, it can investigate the development and evolution of this area. A similar operation was applied to the authors; (2) co-authorship analysis on the units of authors and countries: by showing the network map to dig out collaborations among authors and countries; (3) citation analysis on documents: to list the most cited records; (4) co-occurrence analysis of keywords: referring to two or more keywords appearing in one article, and through data cleaning, keywords with over 100 frequencies were structured into networks, overlaps, and density visualizations to comprehensively explore the current research frontiers and prospects. Data cleaning was also conducted via VOSviewer, where original data exported in “txt” files were loaded to perform a “co-occurrence” analysis of “all keywords” with a minimum of 1, and the map was saved in the “csv” format to get a whole list of all 6,738 keywords. By looking through the “csv” file, items with identical meanings but in different expressions, referring to a pair of keywords that differ by a hyphen like “lung-cancer” and “lung cancer,” or emerge in singular and plural form, respectively, like “association” and “associations,” were picked out from a thesaurus file, where there were two columns named “label” and “replace by.” Moreover, meaningless keywords like “(25)” and “5” were dismissed from analysis with the help of this thesaurus file. Filtered by this file, for example, the frequency of “lung cancer” (*n* = *a*) in the “label” column was calculated together with “lung cancer” (*n* = *b*) in the “replace by” column, and finally, there appeared the keyword “lung cancer” (*n* = *a* + *b*; Supplementary Data). After that, while creating the map, keywords appearing in the search strategy, including “fine particulate matter,” “PM_2.5_,” and “cancer,” were deselected in the step of “verify selected keywords,” which meant they were excluded from the analysis to reduce bias (15).

In the present study, CiteSpace was operated by importing the WOS data, setting the timespan to “2012–2021” (slice length = 1 year), selecting the “keyword” node type, and others following the default. When the map tended to be stable, “Burstiness,” “Refresh,” and “View” on the control panel were clicked, and “20” was entered to identify the top 20 keywords with the strongest citation burst.

HistCite provided the statistical results of citations in the local database. SPSS version 26.0 was used to analyze the correlation between the publication year and annual publications.

## Results

### Analysis of annual publications and average citations per year

**Figure 1:**
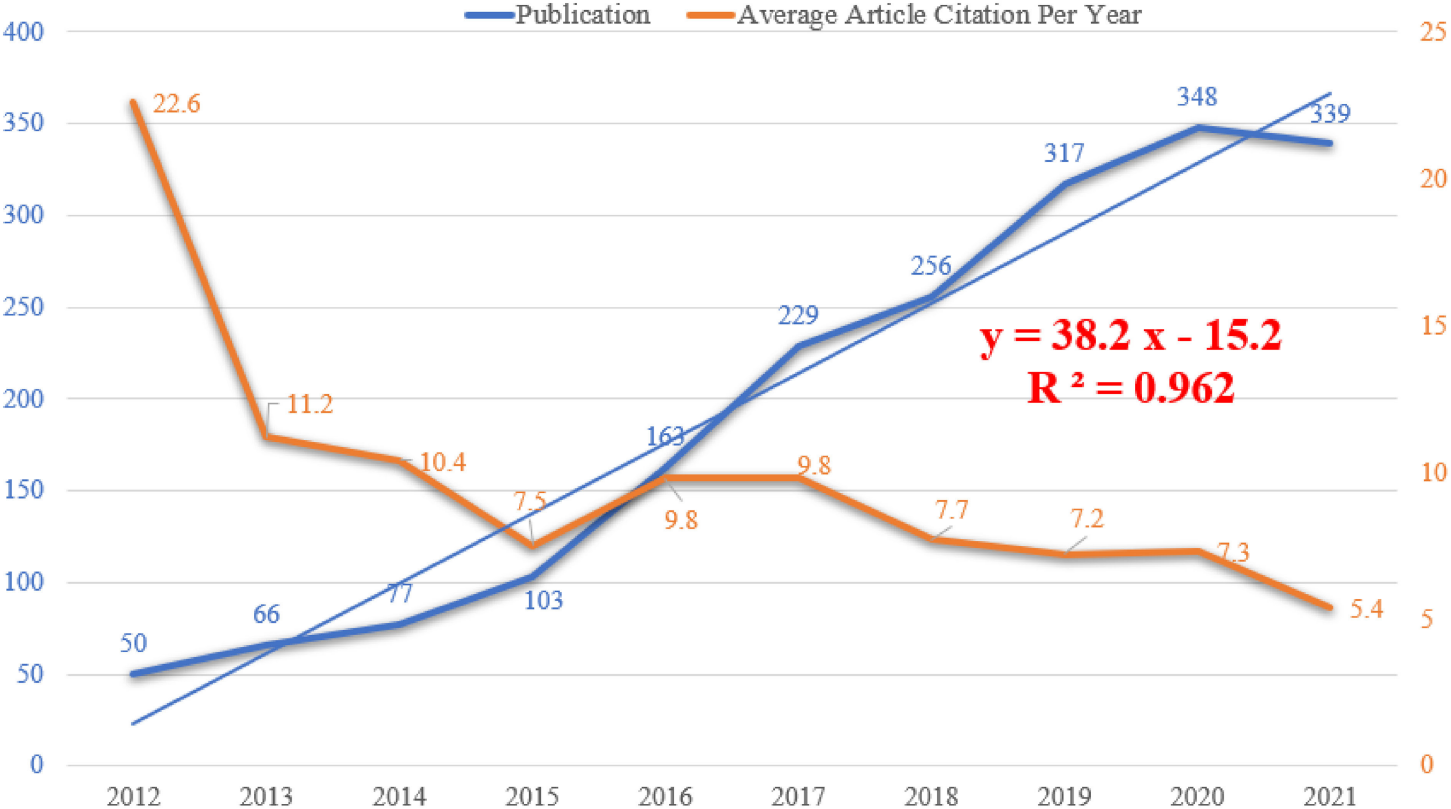
Temporal changes of the number of publication and average citation per year.

Annual publications provided an overview of the field of fine particulate matter and cancer, and the specific number of annual publications from January 2012 to December 2021 has been correspondingly displayed in Figure 1. On the whole, scientific production increased from 50 per year (2.6%) in the first year to 339 per year (17.4%) in 2021, with a slight turning point in 2020 when the 348 scientific productions reached their peak for the decade. Although the growth rate differed as time went by, it can be divided into three stages. From 2012 to 2014, the yearly outputs rose moderately, with a mean of 24.3%. The next 3 years witnessed a duplicated trend of 44.2%. However, from 2017 on, the percentage increase in annual exports was observed to decline as follows: 11.8% (2017), 23.8% (2018), 9.8% (2019), and −2.7% (2020). Overall, the increasing and fluctuating count of publications reflected the importance of the domain between fine particulate matter and cancer. The fitting curve drawn by Microsoft PowerPoint 2019 described a linear growth trend (*y* = 38.3*x* – 15.2, *R*^2^ = 0.962), revealing a significant correlation (*p* < 0.01) between the number of annual publications and the publication year. Analysis of the temporal change of citation was also a necessity to appraise the scientific impact of the publications (16). The curve in orange showed a fluctuant tendency in average article citations per year, ranging from 5.4 to 22.6. The top 3 years were 2012 (22.6), 2013 (11.2), and 2014 (10.4).

### Analysis of authors and institutions

**Figure 2:**
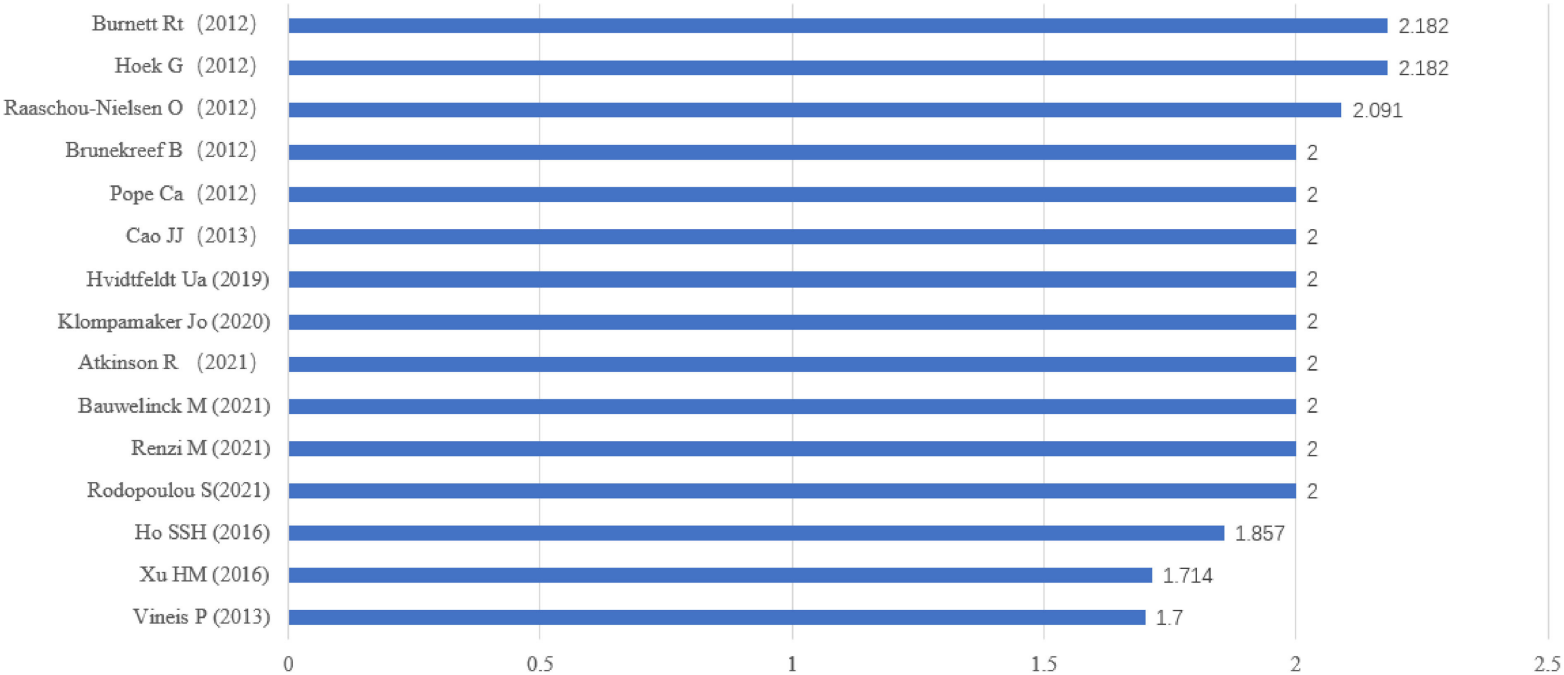
Top 15 authors in M index order enclosed the first publication year.

About 10,000 academic researchers have made important contributions to the study of PM_2.5_ and cancer. Professor Ole Raaschou-Nielsen, from the Department of Environmental Science, Aarhus University, the same university as Brandt J and Ketzel M, published the most papers, with 39 publications. Gerard Hoek (33), Richard T. Burnett (30), Cao JJ (27), Bert Brunekreef (26), C. Arden Pope III (26), Kees de Hoogh (25), van Donkelaar A (24), Brandt J (21), and Ketzel M (21) were also active in this field. Among them, Professor Cao Junji was the head of Chinese academic workers in this field and was the only author from China. He ranked higher in the number of publications than in the citations (Supplementary Table S1). Gerard Hoek, together with Richard T. Burnett, earned the highest H index (24; Supplementary Table S1) and M index (2.182, 2012; Figure 2). M index, the H index divided by the count of years since academic work has been active in this field, which covers the shortage of the H index, as the latter is unsuitable for comparing scientists within the same field with different career spans, serves as a proxy for productivity and the impact of an author (14). The graph consisting of 15 authors in the M index order revealed Professor Atkinson Richard, Bauwelinck Mariska, Rodopoulou Sophia, and Renzi Matteo as impactful newcomers to this realm with an M index of 2 (Figure 2). C. Arden Pope III, Mary Lou Fulton Professor of Economics at Brigham Young University, ranked fifth in productivity but ranked first in total citations (13,925) and local citations (742; Supplementary Table S1).

Those who wrote more than 10 documents were further analyzed. After the exclusion of six items that were not connected, the network map characterized the collaboration of 76 entities sorted into six clusters (Supplementary Figure S2). Professor Brunekreef Bert had the most links (50), followed by professor Ole Raaschou-Nielsen (42), who had the strongest links strengths reaching up to 471. Labels in the red and yellow clusters, respectively, led by Brunekreef B and Raaschou-Nielsen O, represented dense interactions not only within the separate cluster but also between the two clusters, while the purple cluster with the head of Cao JJ from China witnessed more communications just within the cluster. It can be inferred that China tended to share more domestic collaboration than global collaboration compared to other countries.

Using VOSviewer to conduct a co-citation analysis on authors, the top 10 most co-cited authors were listed in Supplementary Table S2. Pope Ca and Raaschou-Nielsen O appeared on the list with 1,056 and 390 publications, respectively, and were also considered prolific authors, as shown in Supplementary Table S1.

With regard to related organizations, the Chinese Academy of Sciences was the most fervent institution, and Utrecht University was the most cited one. As illustrated in Supplementary Table S3, five organizations were from China, two were in the USA, one was in Denmark, one was in Canada, and one was in the Netherlands, which constituted the top 10 prolific institutions. Except for the Chinese Academy of Sciences and Health Canada, the majority of the aforementioned 10 affiliations were universities. Aarhus University won eighth place with 45 publications, with the most prolific author being Professor Raaschou-Nielsen O, who contributed 39 publications.

### Analysis of journals

Through data analysis, the top 10 productive journals are listed in Table 1. With 123 documents, *Science of the Total Environment* was the most productive journal, while seven of the 10 journals produced more than 50 outputs. Apart from the count of papers, the impact factor (IF) is another accepted metric to gauge the importance of journals within the same area, referring to the ratio of the number of citations the journal receives to the number of total citable publications over the last two years, which can be queried by Journal Citation Reports (JCR) in the Web of Science database. The 2022 impact factor of these 10 journals varied from 4.530 to 13.352. Among them, *Environmental International* was the highest, while *Aerosol and Air Quality Research* was the lowest. The more impact factors a journal possessed, the more popular it was in its field. Of 10 journals, *Environmental International, Science of the Total Environment*, and *Environmental Health Perspectives* had an IF of over 10. Three journals' IF lay in the interval of 7–10, containing *Environmental Pollution* (9.988), *Environmental Research* (8.431), and *Ecotoxicology and Environmental Safety* (7.129). The other four journals had an IF between 4 and 6: *Aerosol and Air Quality Research* (4.530), *International Journal of Environmental Research and Public Health* (4.614), *Environmental Science and Pollution Research* (5.190), and *Atmospheric Environment* (5.755). According to the impact factor, the journals were divided into different partitions from Q1 to Q4, each accounting for 25%. According to the JCR partition analysis, almost all the top 10 journals are in Q1 except for *Aerosol and Air Quality Research* and *Environmental Science and Pollution Research*. Judging from the above discussions, *Science of the Total Environment* and *Environmental International* occupied the core status, as the former generated the most articles with an IF over 10. The latter had the highest IF and was in third place in terms of productivity.

**Table 1:** Top 10 productive journals with IF and JCR partition.

**Rank**	**Journal**	**Publication**	**2022 impact factor**	**2022 JCR partition**
1	*Science of the Total Environment*	123	10.753	Q1
2	*Environmental Pollution*	98	9.988	Q1
3	*Environmental International*	86	13.352	Q1
4	*Environmental Science and Pollution Research*	85	5.190	Q2
5	*International Journal of Environmental Research and Public Health*	79	4.614	Q1
6	*Environmental Research*	70	8.431	Q1
7	*Atmospheric Environment*	65	5.755	Q1
8	*Ecotoxicology and Environmental Safety*	51	7.129	Q1
9	*Aerosol and Air Quality Research*	49	4.530	Q2
10	*Environmental Health Perspectives*	45	11.035	Q1

### Analysis of countries

Overall, 90 countries were involved in the research involving fine particulate matter and cancer. The distribution of these countries is depicted in Supplementary Figure S3. America, Asia, Europe, and Austria contributed to the majority of scientific productions. It also suggested that many countries were paying attention to this field, as so many countries turned blue on the world map. As shown in Supplementary Figure S4, China ranked first with 915 outputs out of the top 10 productive countries, followed by the USA (542), England (143), India (110), Canada (108), Italy (99), South Korea (79), Germany (76), the Netherlands (71), and France (71). Three were developing countries, yielding 56.7% of documents, and China was responsible for 82.9% of the total of 1,104 publications. In terms of citations, the USA ranked first with 35,880 citations, followed by China (34,727), England (20,230), Canada (17,590), and France (15,602), among which four out of five were developed countries. China, India, and South Korea were three developing countries that ranked first, fourth, and seventh in publications but ranked second, eighth, and ninth in citations out of 10, respectively.

**Figure 3:**
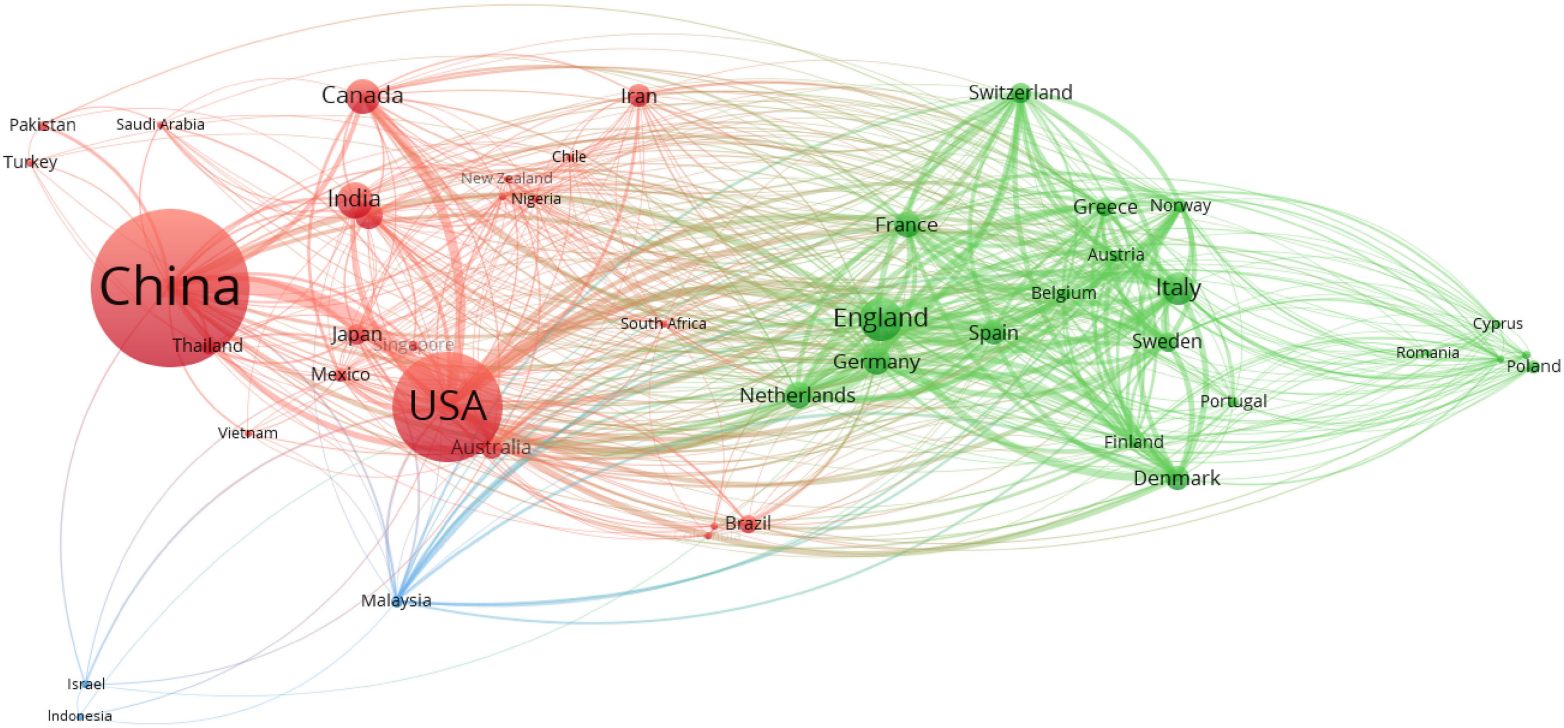
Network map of countries.

To visualize the collaboration between countries, VOSviewer was utilized, where a total of 46 countries were drawn to meet the threshold of a minimum of five publications. According to the network map, the larger a node was, the more articles a country produced, while the thickness of lines between nodes indicated the strength of collaboration (Figure 3). Therefore, presented by the node of the biggest size, China became the most prolific among recruited countries. With the highest total link strength of 703, the USA collaborated substantially with other countries, especially China.

### Analysis of keywords

**Figure 4:**
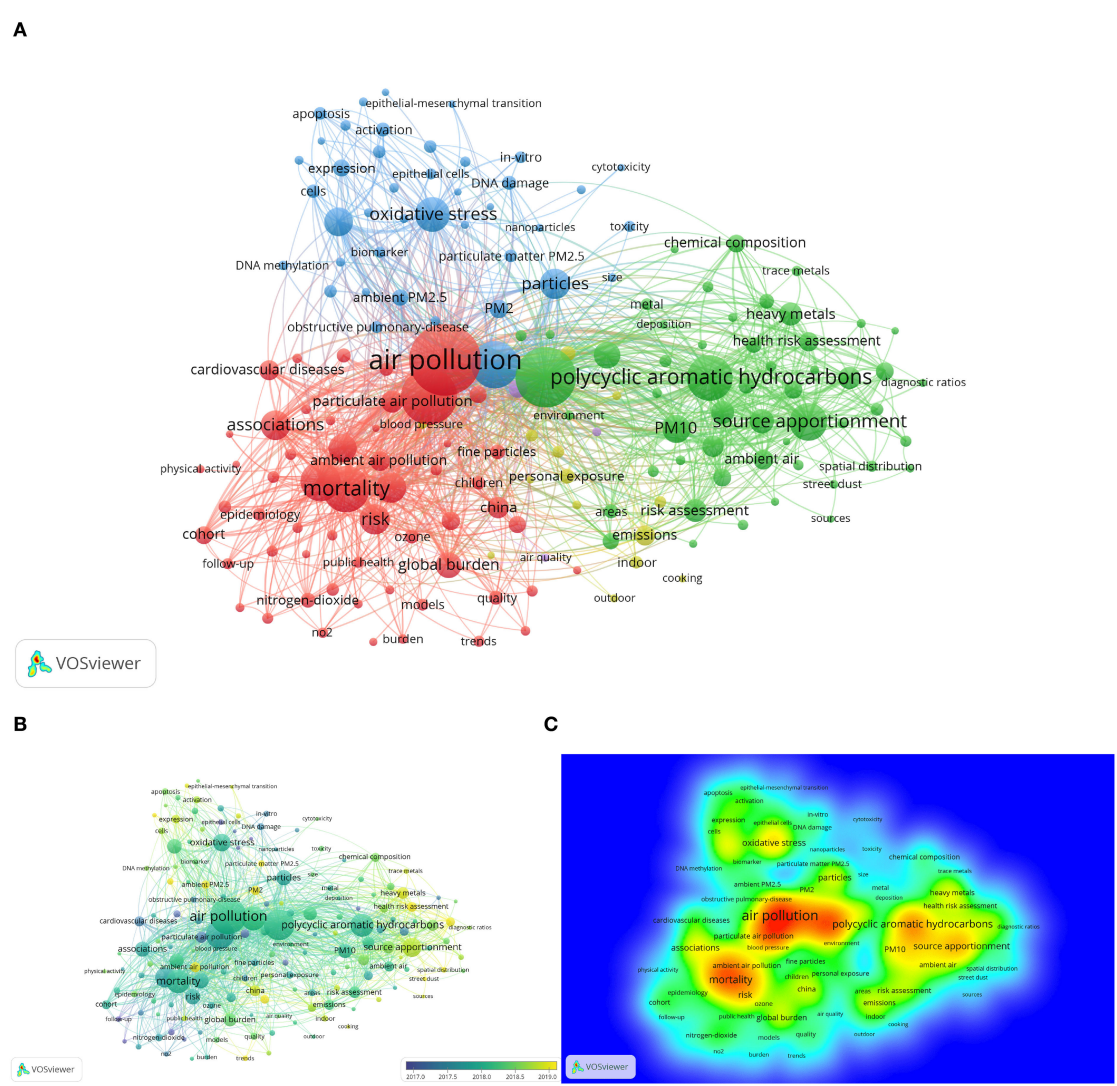
**(A)** Co-occurrence analysis of keywords in frequency. **(B)** Overlap visualization of keywords. **(C)** Density map of keywords.

As summaries of research topics, keyword analysis can reflect the frontiers of the research domain and indicate the future direction. Among 6,738 keywords, including author keywords and keywords plus, due to space limitations and for brevity, filtered by a thesaurus file, frequencies of synonyms were calculated together, such as “lung cancer” and “lung-cancer.” Meanwhile, the major search terms “fine particulate matter,” “PM_2.5_,” and “cancer” were excluded from the analysis to reduce bias. Afterward, searches with over 20 occurrences were extracted. According to the aforementioned criterion, VOSviewer identified 169 keywords that formed five clusters (Figure 4A). The top five keywords in frequency order were “air pollution” (*n* = 797), “particulate matter” (*n* = 612), “lung cancer” (*n* = 531), “exposure” (*n* = 405), and “mortality” (*n* = 398). Cluster 1 in red mainly covered “air pollution,” “lung cancer,” “mortality,” “associations,” “risk,” “health,” and “disease.” Cluster 2 in green included “particulate matter,” “polycyclic aromatic hydrocarbons,” “source apportionment,” “chemical composition,” “heavy metals,” “aerosols,” “city,” and “urban.” Cluster 3 in blue primarily comprised “exposure,” “oxidative stress,” “inflammation,” “DNA damage,” and “expression.” Cluster 4 in yellow constituted “personal exposure,” “emissions,” “indoors,” and “outdoors,” while cluster 5 in purple only contained “fine,” “human health,” and “air quality.” To sum up, cluster 1 mirrored the researchers' attention to the impacts of fine particulate matter on humans. Cluster 2 investigated the components and sources of PM_2.5_. Cluster 3 indicated they were interested in the potential mechanisms of the above influences. Cluster 4 discussed under what circumstances PM_2.5_ may pose a threat to the health of the public, which can be seen as a sub-theme of Custer 2. Cluster 5 emphasized the feature of PM_2.5_, which is tiny enough to travel into the human respiratory tract and affect human health, which was a premise of cluster 1.

As part of the co-occurrence analysis of keywords, VOSviewer also generated an overlap visualization, the color of which suggests the average publication year of the selected keywords (Figure 4B). It can be concluded that most of the keywords were published in 2018 with greener or yellower appearances. “Air pollution,” “lung cancer,” “mortality,” “polycyclic aromatic hydrocarbons,” and “source apportionment” formed a red region in the density map owing to their higher occurrences, while keywords with lower frequencies turned out to be green instead (Figure 4C).

**Figure 5:**
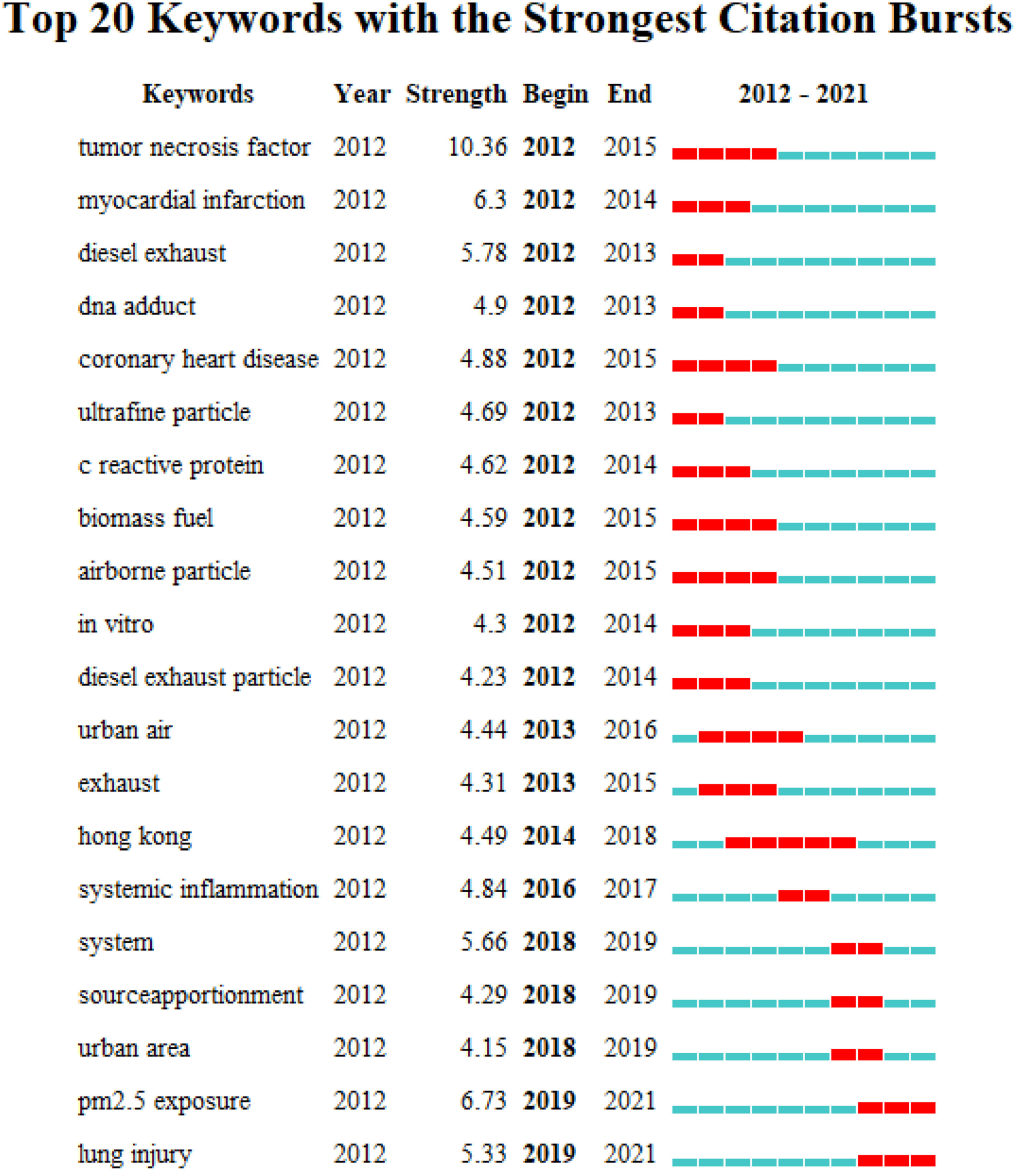
Top 20 keywords burst map by CiteSpace.

To depict the evolutionary pathway of hotspots, a keyword burst map of the top 20 keywords with the strongest citation burst was displayed in Figure 5 by CiteSpace. From 2012 to 2021, “tumor necrosis factor,” “myocardial infarction,” “diesel exhaust,” “dna adduct,” “coronary heart disease,” “ultrafine particle,” “c reactive protein,” “biomass fuel,” “airborne particle,” “*in vitro*,” “diesel exhaust particle,” “urban air,” and “exhaust” started to attract attention in the early years (2012–2014), and in the middle stage (2015–2017) “systematic inflammation” and “hong kong” came into the spotlight. Regarding the latest phase (2018–2021), “system,” “source apportionment,” “urban area,” “PM_2.5_ exposure,” and “lung injury” have become hot topics.

### Analysis of the documents

With a total of 7,018 citations in the Web of Science database, the article, whose first author was Stephen S Lim, published in the *Lancet* in 2012, was regarded as an influential work for holding the highest total citation among the retrieved 1,948 documents and, expectedly, had the largest total citation per year with an average of 631.82. In the local dataset, the review “Estimates and 25-year trends of the global burden of disease attributable to ambient air pollution: an analysis of data from the Global Burden of Diseases Study 2015” by Cohen AJ et al. achieved the highest number of local citations with a count of 153. The distribution of citations revealed that the count of publications decreased as citations rose (Supplementary Figure S5). Notably, the 1,653 records' citations ranged from 0 to 50, among which 61 documents that had not yet been cited accounted for 3.7%. Ten publications with over 500 citations existed in the global dataset, and 37 outputs had been cited more than 200 times. Among the 10 most cited publications, four of the papers were in the *Lancet*. Considering that citations increased with time, surprisingly, only three of the top ten studies were published in 2012, which ranked first, seventh, and eighth. The publications were written by Lim SS, Eeftens M, and Lepeule J, respectively.

In the lists of top ten global and local most cited documents, the only two reviews were those by Xing YF in 2016 and Hamra GB in 2014. There were a total of 104 reviews of 1,948 records, so the proportion of reviews in all document types (5.3%) among all retrieved publications was nearly half that among the 10 most-cited global documents (10%; Supplementary Tables S4, S5).

**Figure 6:**
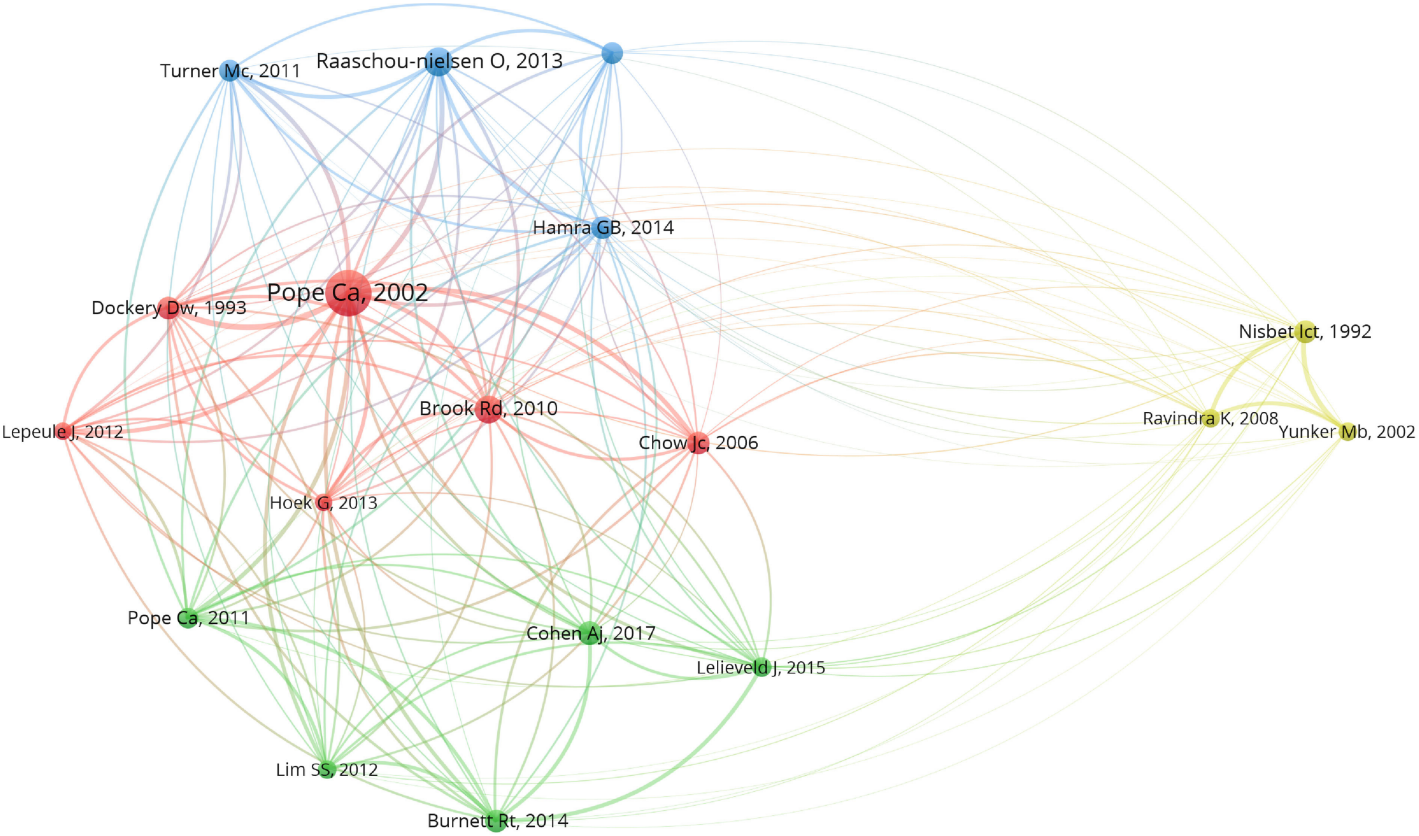
Network map of 18 most co-cited references.

Of all 61,554 cited references in the local dataset, 18 documents were deemed to be infusive works and intellectual foundations of this field with over 100 co-citations, which formed four clusters when analyzing the co-citation relationship (Figure 6). Each label contained the first author and publication year of the article. Cluster 1 in red demonstrated the effects of long-term exposure to fine particulate matter due to air pollution on health, especially as a risk factor for lung cancer or cardiovascular mortality. Evidence also emphasized the positive association between PM_2.5_ and lung cancer (17). The biggest bubble was created by Pope Ca, 2002, which was the most co-cited reference with 397 citations and 798 total link strength. Cluster 2 in green was centered on assessing the global burden of diseases attributed to exposure to ambient particle matter, among which the Cox proportional-hazards model was most frequently utilized (18). Burnett et al. (19) made improvements to this model by integrating RR information from various combustion sources to be a predictor of the leading cause of mortality due to air pollution. Cluster 3 in blue was made up of studies discussing the carcinogenicity of PM_2.5_, which was associated with increases in genetic damage, including altering gene expression, evoking mutations in cells, whether somatic or germinal, and inducing cytogenetic abnormalities (20, 21). Cluster 4 in yellow primarily focused on the source apportionment, constituents, and toxicity of polycyclic aromatic hydrocarbons, which are commonly bound in PM_2.5_ (22). It further explained the link between emissions and exposure. As the health influences of PM_2.5_ on humans vary according to what is absorbed in PM_2.5_, it is essential to distinguish the sources of pollutants to significantly reduce air pollution.

## Discussion

A bibliometric study was performed focusing on the relationship between PM_2.5_ and cancer. Among the 1,948 enrolled documents, a total of 9,796 academic workers were involved in this field, publishing in 484 journals for 2,490 organizations in 90 countries. The main findings were as follows:

(1) The research revealed a positive trend in the production of PM_2.5_ and cancer over the 10-year analysis period. 2012 was the year that had the highest average number of citations per year.
(2) Among the 10 most prolific authors, Raaschou-Nielsen O and Pope Ca played crucial roles; the former was the most productive author, and the latter was the most cited academic researcher, whether in the Web of Science database or the local dataset, and was also the most co-cited author. Gerard Hoek and Richard T. Burnett were also in the spotlight, with the highest H and M indices. Cao JJ, the only academic from China in the top ten, ranked higher in the number of publications than in the number of citations. Cluster visualization revealed that, compared to experts from other nations, Chinese scholars were more likely to collaborate within their own country.
(3) Chinese Academy of Sciences was the most fervent institution, and Utrecht University was the most cited one. Universities played an important role in the number of publications, and they were the prominent backbones as eight out of the 10 top productive institutions were colleges.
(4) Led by China and the USA, which generated 74.8% of publications, these two countries also witnessed the strongest collaborative linkage, but as the most productive country, the average citation of a publication from China accounted for four-sevenths of that from the USA. Further, the developing countries of the 10 had a lower rank in citations than in publications. To sum up, authors, organizations, and country analyses presented that China had a lower citation per publication because it did contribute to scientific productions but owned fewer citations.
(5) *Science of the Total Environment* and *Environmental International* took over the central situation because the former possessed an IF of more than 10 as the most productive journal. The latter enjoyed the highest IF and was in third place for productivity. Ten leading journals mostly lay in Q1, and their IF in 2022 ranged from 4.53 to 13.352.
(6) The analysis of keywords and co-cited references can be integrated and sorted into four main research categories. First, it elucidated the health effects of chronic exposure to PM_2.5_ from air pollution, concentrating mainly on its positive correlation with lung cancer, as “air pollution,” “lung cancer,” “mortality,” “associations,” “risk,” “health,” and “disease” were the leading keywords. In all the literature, the concentration of PM_2.5_ ranged from less than 10 μg/m^3^ to more than 100 μg/m^3^ (21). Numerous studies concluded that every 10 μg/m^3^ increase resulted in an increased risk of lung cancer (23, 24). Even exposure to PM_2.5_ concentrations below the limit showed a positive association between PM_2.5_ and cancer. No safe level of PM_2.5_ has been observed until now (25).

Given the damage caused by PM_2.5_ and its status as the second-most polluted country, China has been monitored for its increasing concentration of PM_2.5_, which necessitates a reduction (26). An interesting and commonly noted phenomenon was that, compared to non-smokers, smokers' susceptibility to lung cancer mortality from PM_2.5_ was observed as less significant (27). According to Harma, it may be because polycyclic aromatic hydrocarbons existent in different sources like tobacco smoke, PM_2.5_, and diesel exhaust can compete for metabolic activation (20); however, the exact mechanism remains unclear, which warrants subsequent exploration. Other than lung cancer, a time-series study in Xi'an found a significant association between PM_2.5_ and mortality from the stomach and colorectal cancer (28). A meta-analysis was carried out in 2021 to show that fine particulate matter was related to the increased risk of hepatocellular carcinoma. Alanine aminotransferase acted as a mediator between PM_2.5_ and HCC (29). On account of its tiny size, PM_2.5_ can travel into the human body by respiration or ingestion and then have access to blood (8), suggesting that PM_2.5_ exposure was significantly associated with several cancer-specific mortalities, including those of the oral cavity and nasopharynx, esophagus, stomach, colon rectum, liver, gallbladder, larynx, lung, bone, skin, female breast, cervix, prostate, brain, and leukemia (12). Therefore, “fine,” “human health,” and “air quality” were included. “Children” was also a keyword because PM_2.5_ promoted the progression of the most common cancer in this population, leukemia (8). Studies on the incidence and mortality of lung cancer attributed to PM_2.5_ have been discussed at length. As “lung cancer” (*n* = 531) occurred at the third highest frequency, the past few years have witnessed a drift from lung cancer to other types, such as “breast cancer” (*n* = 36).

The second focus, represented by “particulate matter,” “polycyclic aromatic hydrocarbons,” “source apportionment,” “chemical composition,” “heavy metals,” “aerosols,” “city,” and “urban,” was about the sources and composition of PM_2.5_. These studies aimed to identify the carcinogenicity of each component and its potential pathway, involving increased cancer risk. Therefore, air pollution can be controlled from sources (20). “Polycyclic aromatic hydrocarbons” was most commonly carried on the particulate matter, followed by “heavy metals” and so on. A study set in the cities of Karaj and Fardis revealed that PM_2.5_ could suppress the activity of mitochondrion in A549 cells with high concertation of polycyclic aromatic hydrocarbons and heavy metals Zn, Fe, Pb, and Cu (30). This phenomenon was more significant in cold seasons like autumn and winter and in areas with heavy traffic, which may account for “city” and “urban” showing up as keywords. A study in Shanxi Province, China, implied combustion emissions were the largest contributors to cancer risk, while cities in the USA emitted most PM_2.5_ from vehicles (31), so the major sources of PM_2.5_ may vary greatly by region, and the effects of PM_2.5_ exposure can differ by season, which appeals for policies suiting the local conditions, like implementing policies to curb emissions. A large body of evidence has pointed to the dangers of PM_2.5_ from outdoor air pollution, but as people actually occupy more time on indoor activities like school and recently have to work from home due to the COVID-19 pandemic, “indoor air pollution” appeared as a keyword is gradually drawing more interest (32); therefore, so under what circumstances that PM_2.5_ may pose a threat to the health of the public including “personal exposure,” “emissions,” “indoor” and “outdoor” can be viewed as a sub-focus of the sources and components of PM_2.5_.

The third hotspot, and the most important direction needed for further investigation, was to explain the underlying mechanism of PM_2.5_-induced tumor progression. PM_2.5_ can lead to the excessive production of ROS by activating NADPH oxidase to enhance the expression of inflammatory mediators in leukemia cells through the NF-κB p65 and p-STAT3 pathways (8), while ROS can even cause oxidative stress in lung epithelial cells. Under this condition, cells would undergo apoptosis because proteins, lipids, and DNA have been destroyed (33). Efforts should be made to understand the specific and complex mechanism of one certain PM-related cancer to provide an intervention target. For example, rosiglitazone, an agonist of PPARγ that declined in PM_2.5_-treated A549 cells, was proven to suppress ROS generation and thus be protected from injury when exposed to PM_2.5_ (33). “Exposure,” “oxidative stress,” “inflammation,” “DNA damage,” and “expression” were the keywords of high frequency. To sum up, the possible mechanisms at present are resulting in DNA damage, inducing inflammation, activating the oxidative stress response, altering telomere length, and producing epigenetic changes such as DNA methylation (21). It is necessary to conduct subsequent research on interactions between different mechanisms.

The last frontier referred to developing models for the disease burden caused by particulate exposure. The article published by Lim SS in 2012, with the highest number of citations (7,018), belonged to this area.

In this study, all the figures and tables derived from the software were carefully checked and compared to other reviews. Based on bibliometric methods, our study provided a visual representation of the research process and its frontiers. Despite the above measures, there were still limitations to this research. Rendering standardized records, the Web of Science database was selected as the paramount source of data for bibliometric analysis (10); however, it led to impactful documents in other databases like Scopus being excluded. All the retrieved publications were in one single language; thus, analysis of influential works in languages other than English remained undone, which was a limitation considering the integrity of the data. Since 2022 is not yet over, this research is based on the data from 2012 to 2021; thus, new trends in 2022 are not indicated, and all the findings are merely applied to literature analysis before October 12, 2022. When conducting co-occurrence analysis, there was a step to screen and integrate the keywords with the same meaning, which was indeed a subjective process.

## Conclusion

In this study, with the assistance of bibliometric software such as VOSviewer, CiteSpace, Biblioshiny, and HistCite, literature retrieved from the Web of Science database was analyzed. The temporal change of publications and the characteristics of publications, like the leading authors, journals, institutions, countries, keywords, and citations, were enumerated, and the collaboration visualization was delineated. Exposure to PM_2.5_ for a long time was positively associated with cancer risk, and the toxic mechanism needs more investigation. By identifying the compounds in PM_2.5_, it appeals to the public to raise awareness and experts to explore and exploit cleaner energy sources. Although many countries have taken action to improve air quality, many regions have PM_2.5_ levels above the limit. As the most prolific publisher and the second-most polluted country with an increasing PM_2.5_ concentration, China still has a long way to go. Even exposure to PM_2.5_ levels below the limit can be detrimental to human health due to different constituents. Therefore, researching this topic remains a long-term process.

## Data availability statement

The original contributions presented in the study are included in the article/Supplementary material, further inquiries can be directed to the corresponding author.

## Author contributions

XL, QY, and DZ were responsible for data analysis and the initial draft. XL revised the second draft according to feedback from HT and LC. JW, ZJ, and YC assisted in a literature search. ZL contributed to the study's conception and design. All authors listed have read, revised, and approved the final version for publication.

## Funding

This work was supported by the Special Fund Project of Guangdong Science and Technology (210728156901524), the Shantou Medical Science and Technology Planning Project (grant numbers 200622115260639, 2021-68-44, and 2022-88-27), and the Administration of Traditional Chinese Medicine of Guangdong Province project (202205092315428030).

## Conflict of interest

The authors declare that the research was conducted in the absence of any commercial or financial relationships that could be construed as a potential conflict of interest.

## Publisher's note

All claims expressed in this article are solely those of the authors and do not necessarily represent those of their affiliated organizations, or those of the publisher, the editors and the reviewers. Any product that may be evaluated in this article, or claim that may be made by its manufacturer, is not guaranteed or endorsed by the publisher.
