# Supplementary material for: A bibliometric and visualization analysis on the association between chronic exposure to fine particulate matter and cancer risk
Source: Front Public Health. 2022 Dec 5;10:1039078. doi: 10.3389/fpubh.2022.1039078 (PMC9762493; doi:10.3389/fpubh.2022.1039078)
Supplement: Supplementary file 1 [file Data_Sheet_1.docx]

Supplementary Material

# Supplementary Data

Enclosed is a thesaurus file used to co-occurrence analysis of keywords.

# Supplementary Figures and Tables

## Supplementary Figures


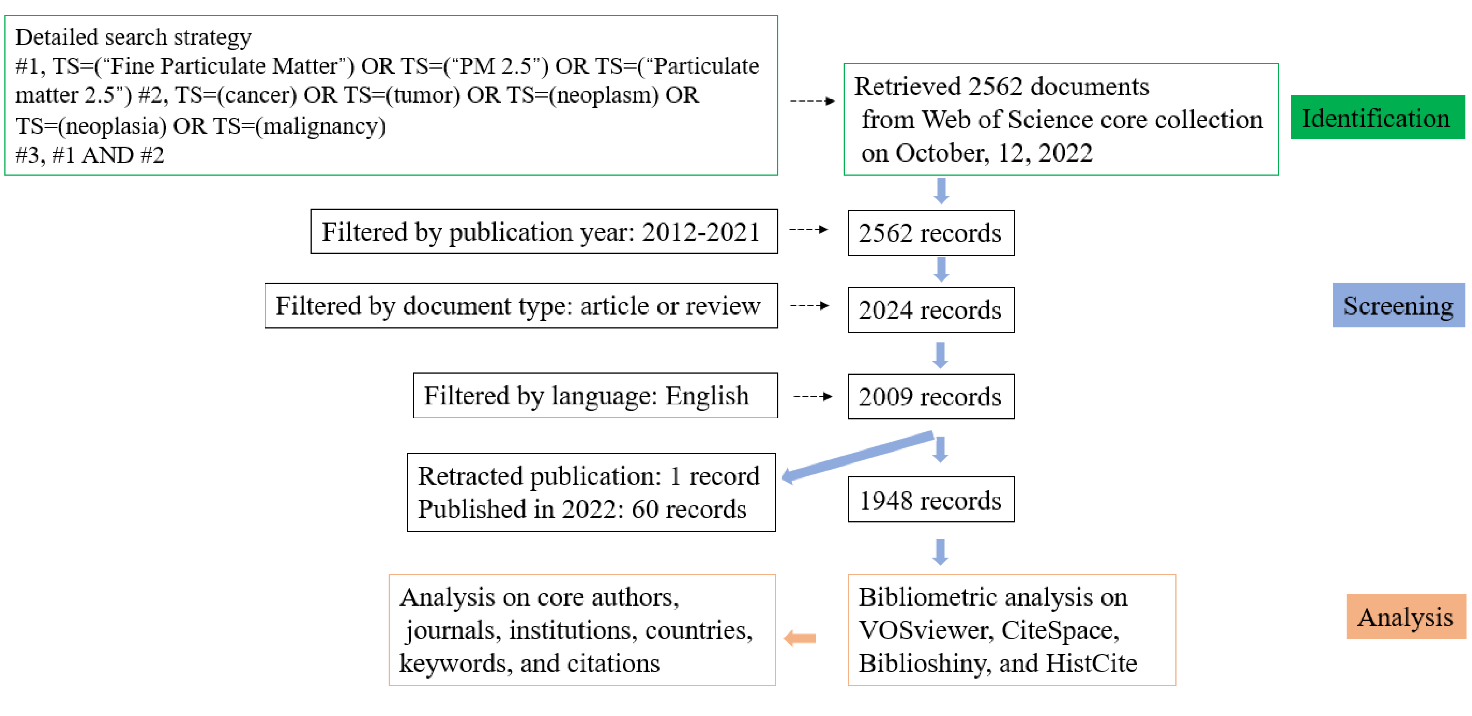
**Supplementary Figure S1.** The literature search and selection process.


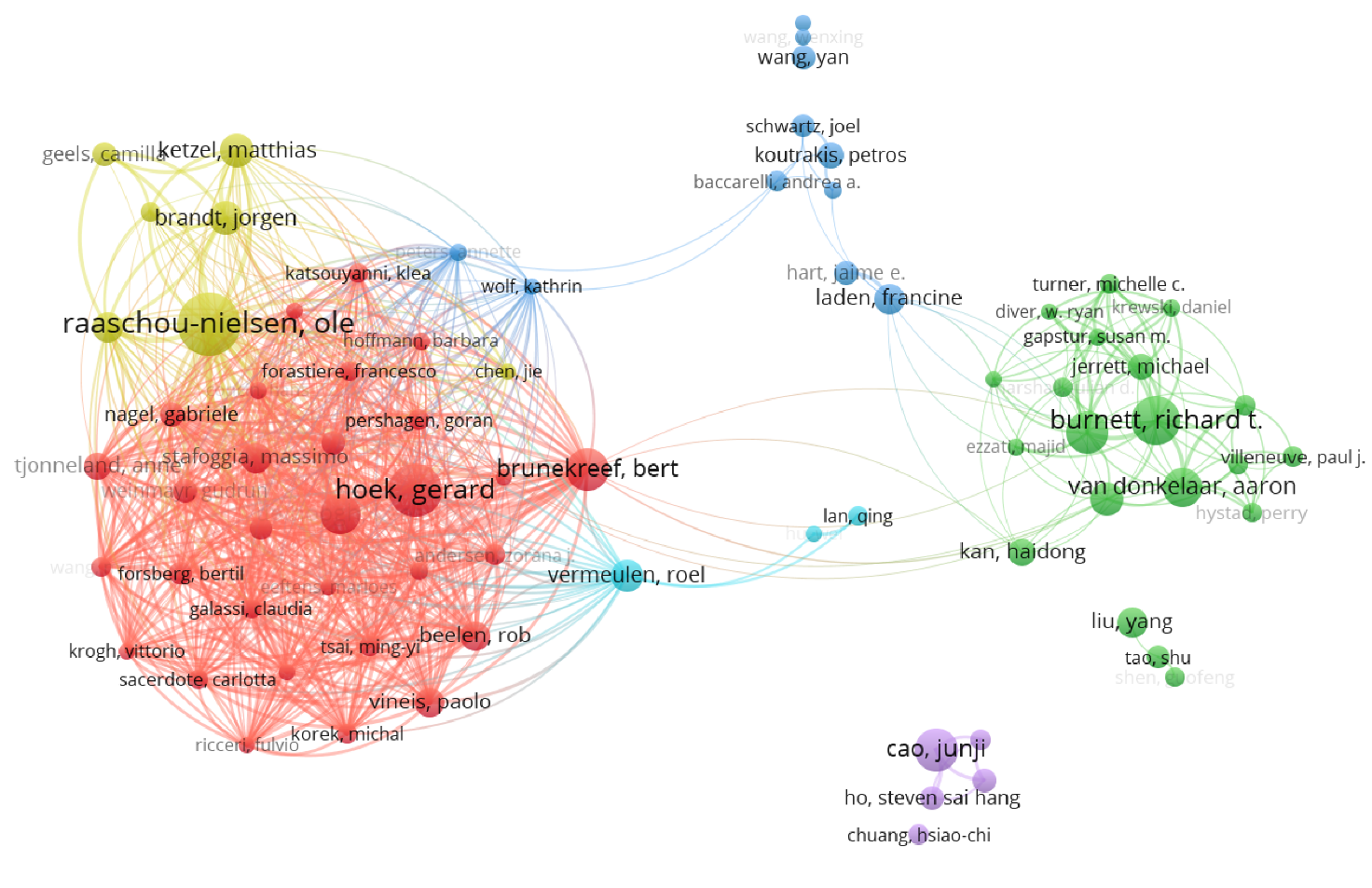


**Supplementary Figure S2**. Network map of authors.


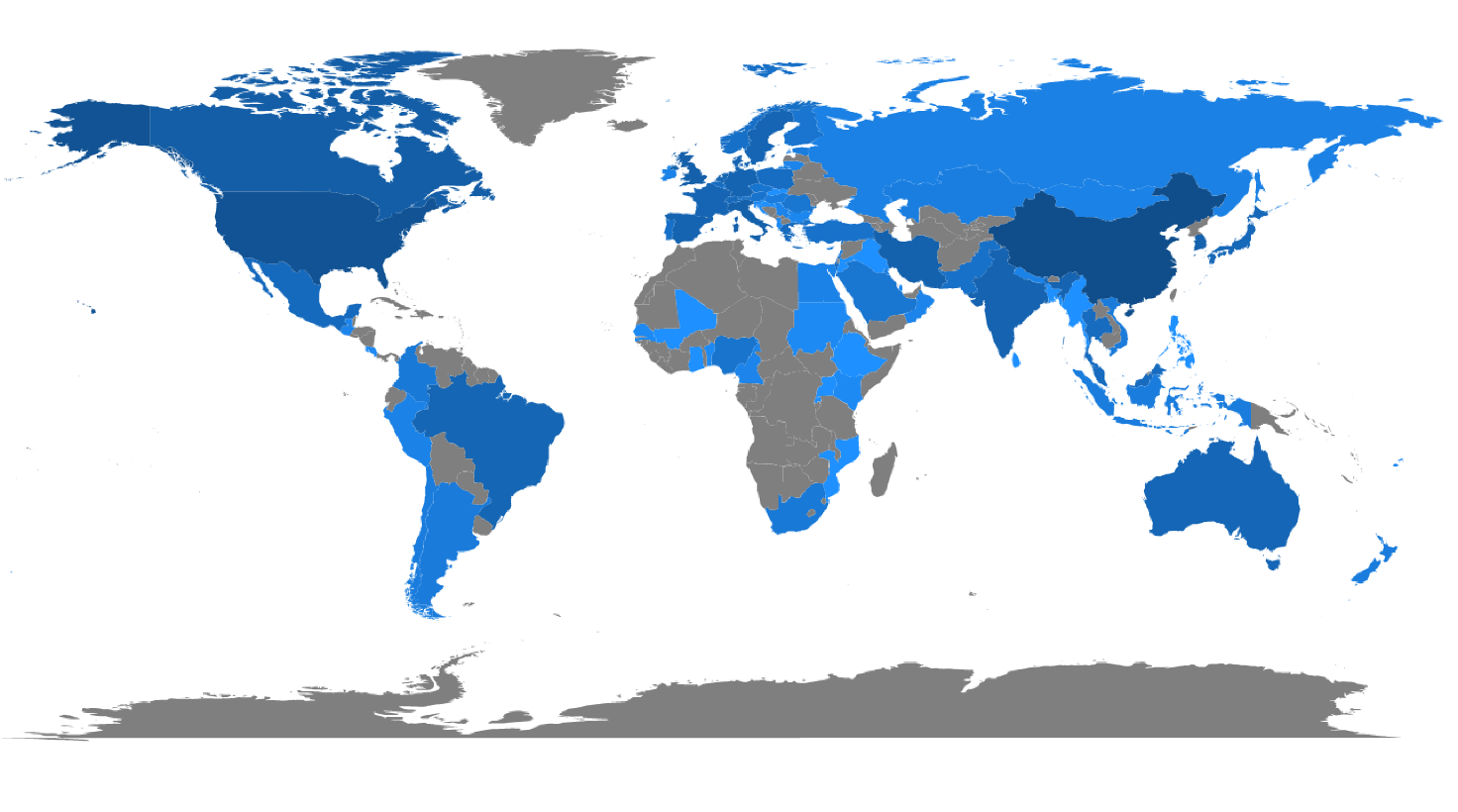


**Supplementary Figure S3**. Distribution of contributed countries.


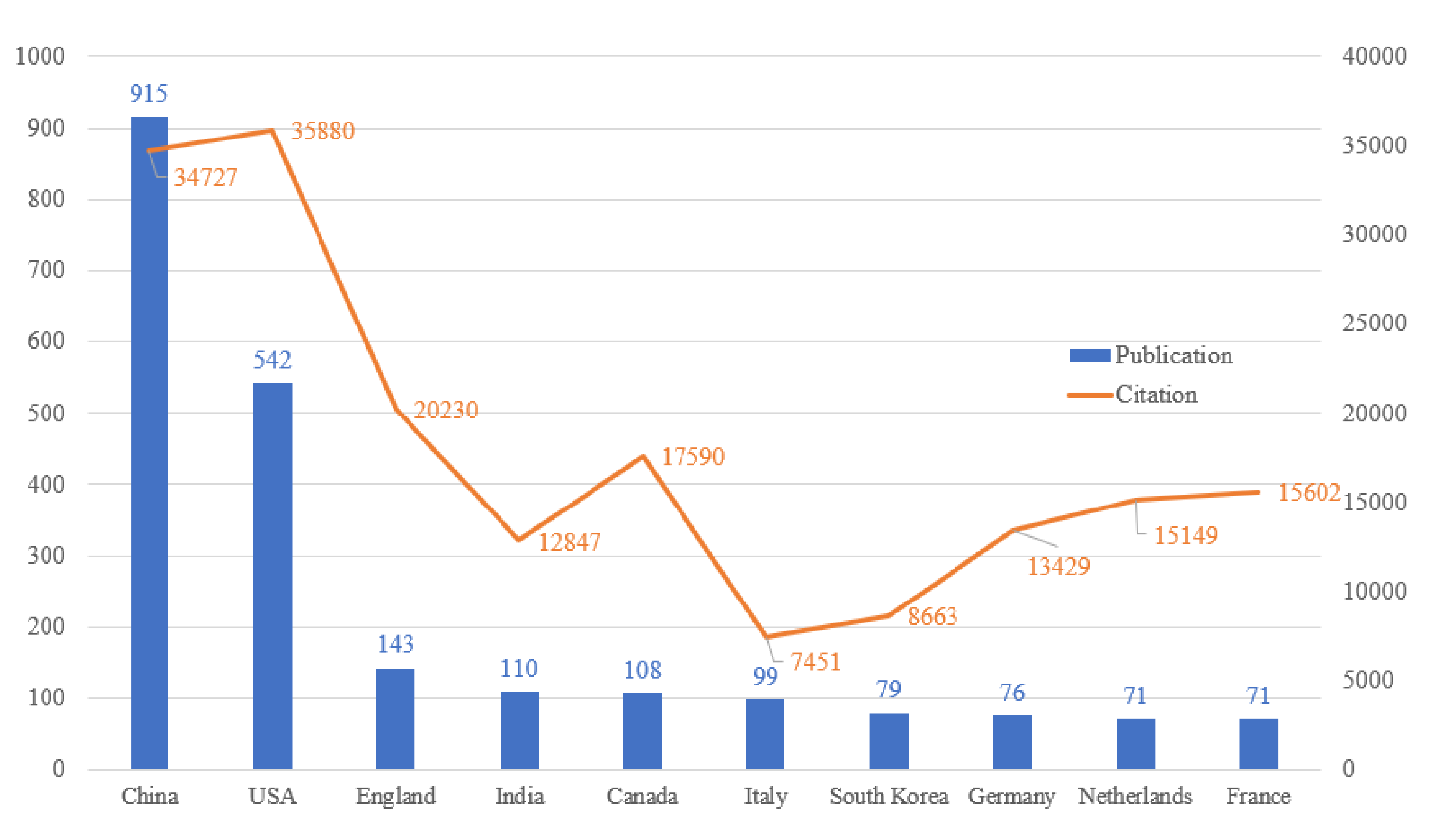


**Supplementary Figure S4.** Top ten prolific countries with citations.


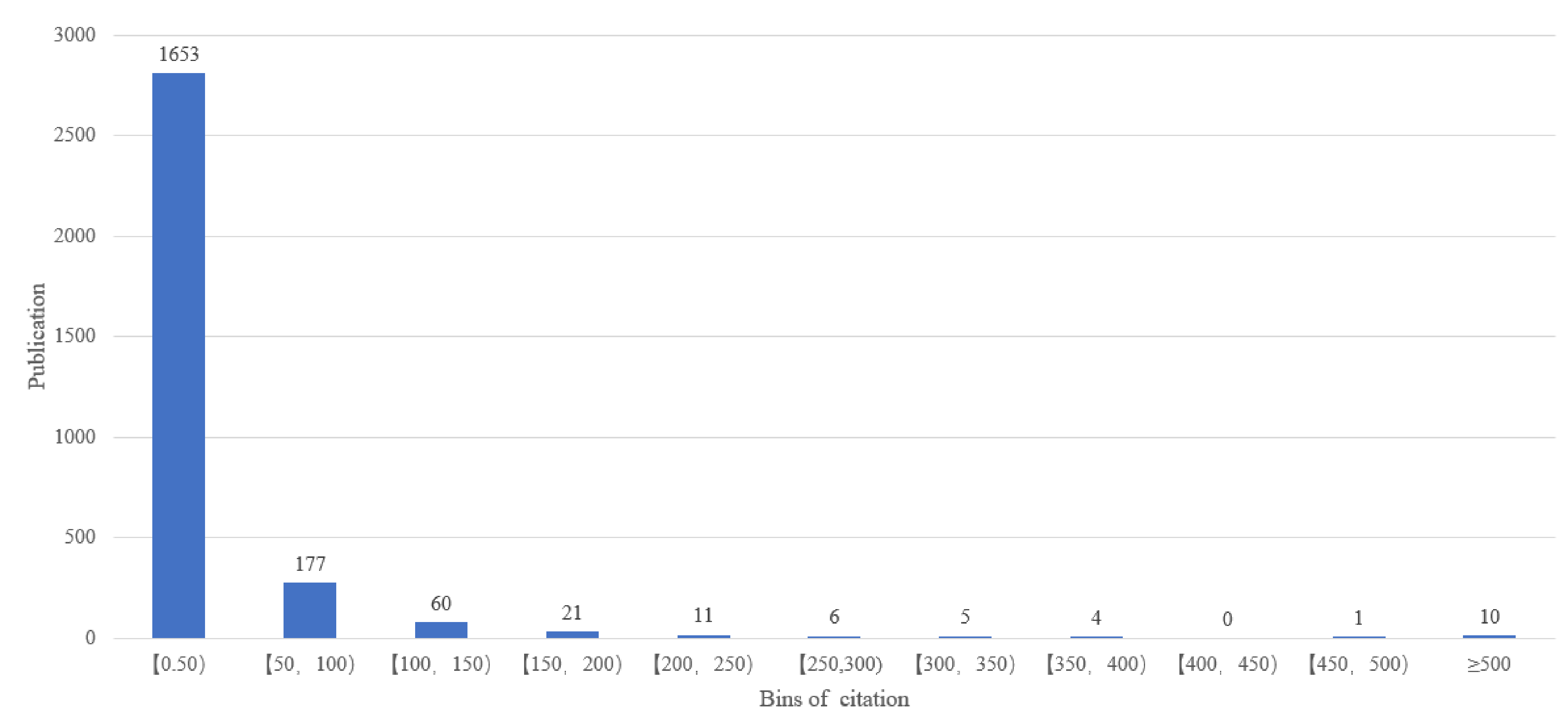


**Supplementary Figure S5.** Distribution of documents based on citations.

## Supplementary Tables

**Supplementary Table S1.** Top 10 productive authors with attached total, local citation and H index.

| Rank | Author | Institution | Publication | Total Citation | Local Citation | H index |
| --- | --- | --- | --- | --- | --- | --- |
| 1 | Raaschou-Nielsen O | Aarhus Univ | 39 | 4428 | 462 | 23 |
| 2 | Hoek G | Univ Utrecht | 33 | 3979 | 323 | 24 |
| 3 | Burnett Rt | Univ Ottwa | 30 | 10859 | 577 | 24 |
| 4 | Cao JJ | Chinese Acad Sci | 27 | 963 | 227 | 18 |
| 5 | Brunekreef B | Univ Utrecht | 26 | 13306 | 550 | 22 |
| 6 | Pope Ca | Brigham Young Univ | 26 | 13925 | 742 | 22 |
| 7 | de Hoogh K | Univ Basel | 25 | 3508 | 281 | 17 |
| 8 | van Donkelaar A | Dalhousie Univ | 24 | 11292 | 514 | 17 |
| 9 | Brandt J | Aarhus Univ | 21 | 389 | 64 | 9 |
| 10 | Ketzel M | Aarhus Univ | 21 | 377 | 64 | 9 |

**Supplementary Table S2.** Top ten most co-cited authors.

| Co-cited Author | Count |
| --- | --- |
| Pope Ca | 1056 |
| WHO* | 686 |
| USEPA* | 565 |
| Raaschou-Nielsen O | 390 |
| Brook Rd | 317 |
| Beelen R | 291 |
| Turner Mc | 256 |
| Cohen Aj | 234 |
| Dockery Dw | 231 |
| Chow Jc | 223 |
| Jerrett M | 206 |

* Referring to a group author.

**Supplementary Table S3.** Top 10 productive institutions and according citations.

| Institutions | Publications | Citations |
| --- | --- | --- |
| Chinese Acad Sci | 129 | 4109 |
| Peking Univ | 73 | 2388 |
| Fudan Univ | 65 | 12673 |
| Univ Utrecht | 57 | 14585 |
| Univ Chinese Acad Sci | 46 | 1705 |
| Univ Washington | 46 | 12991 |
| Aarhus Univ | 45 | 3256 |
| Hlth Canada | 40 | 13971 |
| Harvard TH Chan Sch Publ Hlth | 37 | 989 |
| China Med Univ | 35 | 814 |

**Supplementary Table S4.** Top 10 most cited globally documents.

| Rank | Document | Global Citations | Document Type |
| --- | --- | --- | --- |
| 1 | Lim SS, Vos T, Flaxman AD, Danaei G, Shibuya K, et al.  A comparative risk assessment of burden of disease and injury attributable to 67 risk factors and risk factor clusters in 21 regions, 1990-2010: a systematic analysis for the Global Burden of Disease Study 2010  LANCET. 2012 DEC 15; 380 (9859): 2224-2260 | 7018 | Article |
| 2 | Cohen AJ, Brauer M, Burnett R, Anderson HR, Frostad J, et al.  Estimates and 25-year trends of the global burden of disease attributable to ambient air pollution: an analysis of data from the Global Burden of Diseases Study 2015  LANCET. 2017 MAY 13; 389 (10082): 1907-1918 | 2746 | Article |
| 3 | Burnett RT, Pope CA, Ezzati M, Olives C, Lim SS, et al.  An Integrated Risk Function for Estimating the Global Burden of Disease Attributable to Ambient Fine Particulate Matter Exposure  ENVIRONMENTAL HEALTH PERSPECTIVES. 2014 APR; 122 (4): 397-403  LANCET. 2016 OCT 8; 388 (10053): 1659-1724 | 1076 | Article |
| 4 | Raaschou-Nielsen O, Andersen ZJ, Beelen R, Samoli E, Stafoggia M, et al.  Air pollution and lung cancer incidence in 17 European cohorts: prospective analyses from the European Study of Cohorts for Air Pollution Effects (ESCAPE)  LANCET ONCOLOGY. 2013 AUG; 14 (9): 813-822 | 923 | Article |
| 5 | Beelen R, Raaschou-Nielsen O, Stafoggia M, Andersen ZJ, Weinmayr G, et al.  Effects of long-term exposure to air pollution on natural-cause mortality: an analysis of 22 European cohorts within the multicentre ESCAPE project  LANCET. 2014 MAR 1; 383 (9919): 785-795 | 837 | Article |
| 6 | Xing YF, Xu YH, Shi MH, Lian YX  The impact of PM2.5 on the human respiratory system  JOURNAL OF THORACIC DISEASE. 2016 JAN; 8 (1): E69-E74 | 792 | Review |
| 7 | Eeftens M, Beelen R, de Hoogh K, Bellander T, Cesaroni G, et al.  Development of Land Use Regression Models for PM2.5, PM2.5 Absorbance, PM10 and PMcoarse in 20 European Study Areas; Results of the ESCAPE Project  ENVIRONMENTAL SCIENCE & TECHNOLOGY. 2012 OCT 16; 46 (20): 11195-11205 | 622 | Article |
| 8 | Lepeule J, Laden F, Dockery D, Schwartz J  Chronic Exposure to Fine Particles and Mortality: An Extended Follow-up of the Harvard Six Cities Study from 1974 to 2009  ENVIRONMENTAL HEALTH PERSPECTIVES. 2012 JUL; 120 (7): 965-970 | 614 | Article |
| 9 | Hamra GB, Guha N, Cohen A, Laden F, Raaschou-Nielsen O, et al.  Outdoor Particulate Matter Exposure and Lung Cancer: A Systematic Review and Meta-Analysis  ENVIRONMENTAL HEALTH PERSPECTIVES. 2014 SEP; 122 (9): 906-911 | 566 | Review |
| 10 | Apte JS, Marshall JD, Cohen AJ, Brauer M  Addressing Global Mortality from Ambient PM2.5  ENVIRONMENTAL SCIENCE & TECHNOLOGY. 2015 JUL 7; 49 (13): 8057-8066 | 551 | Article |

**Supplementary Table S5.** Top 10 most cited locally documents.

| Rank | Document | Local Citation | Document Type |
| --- | --- | --- | --- |
| 1 | Cohen AJ, Brauer M, Burnett R, Anderson HR, Frostad J, et al.  Estimates and 25-year trends of the global burden of disease attributable to ambient air pollution: an analysis of data from the Global Burden of Diseases Study 2015  LANCET. 2017 MAY 13; 389 (10082): 1907-1918 | 153 | Article |
| 2 | Hamra GB, Guha N, Cohen A, Laden F, Raaschou-Nielsen O, et al.  Outdoor Particulate Matter Exposure and Lung Cancer: A Systematic Review and Meta-Analysis  ENVIRONMENTAL HEALTH PERSPECTIVES. 2014 SEP; 122 (9): 906-911 | 146 | Review |
| 3 | Burnett RT, Pope CA, Ezzati M, Olives C, Lim SS, et al.  An Integrated Risk Function for Estimating the Global Burden of Disease Attributable to Ambient Fine Particulate Matter Exposure  ENVIRONMENTAL HEALTH PERSPECTIVES. 2014 APR; 122 (4): 397-403 | 144 | Article |
| 4 | Lim SS, Vos T, Flaxman AD, Danaei G, Shibuya K, et al.  A comparative risk assessment of burden of disease and injury attributable to 67 risk factors and risk factor clusters in 21 regions, 1990-2010: a systematic analysis for the Global Burden of Disease Study 2010  LANCET. 2012 DEC 15; 380 (9859): 2224-2260 | 111 | Article |
| 5 | Lepeule J, Laden F, Dockery D, Schwartz J  Chronic Exposure to Fine Particles and Mortality: An Extended Follow-up of the Harvard Six Cities Study from 1974 to 2009  ENVIRONMENTAL HEALTH PERSPECTIVES. 2012 JUL; 120 (7): 965-970 | 100 | Article |
| 6 | Beelen R, Raaschou-Nielsen O, Stafoggia M, Andersen ZJ, Weinmayr G, et al.  Effects of long-term exposure to air pollution on natural-cause mortality: an analysis of 22 European cohorts within the multicentre ESCAPE project  LANCET. 2014 MAR 1; 383 (9919): 785-795 | 87 | Article |
| 7 | Cesaroni G, Badaloni C, Gariazzo C, Stafoggia M, Sozzi R, et al.  Long-Term Exposure to Urban Air Pollution and Mortality in a Cohort of More than a Million Adults in Rome  ENVIRONMENTAL HEALTH PERSPECTIVES. 2013 MAR; 121 (3): 324-33 | 77 | Article |
| 8 | Bandowe BAM, Meusel H, Huang RJ, Ho KF, Cao JJ, et al.  PM25-bound oxygenated PAHs, nitro-PAHs and parent-PAHs from the atmosphere of a Chinese megacity: Seasonal variation, sources and cancer risk assessment  SCIENCE OF THE TOTAL ENVIRONMENT. 2014 MAR 1; 473: 77-87 | 71 | Article |
| 9 | Xing YF, Xu YH, Shi MH, Lian YX  The impact of PM2.5 on the human respiratory system  JOURNAL OF THORACIC DISEASE. 2016 JAN; 8 (1): E69-E74 | 65 | Review |
| 10 | Apte JS, Marshall JD, Cohen AJ, Brauer M  Addressing Global Mortality from Ambient PM2.5  ENVIRONMENTAL SCIENCE & TECHNOLOGY. 2015 JUL 7; 49 (13): 8057-8066 | 62 | Article |
